# Interleukin-1β Gene (*IL1B*) rs1143634 (+3954 C>T) Polymorphism and Cutaneous Melanoma Risk: An Observational Case-Control Study in Northeast Italy

**DOI:** 10.3390/genes17070786

**Published:** 2026-07-09

**Authors:** Sabina Cauci, Cinzia Buligan, Luca Bazzichetto, Gianluca Petris, Giuseppe Stinco

**Affiliations:** 1 Department of Medicine (DAME), University of Udine, Piazzale Kolbe 4, 33100 Udine, Italy; cinzia.buligan@asufc.sanita.fvg.it (C.B.); gianluca.petris@uniud.it (G.P.); giuseppe.stinco@uniud.it (G.S.); 2 Institute of Dermatology, Azienda Sanitaria Universitaria Friuli Centrale (ASUFC), Santa Maria Della Misericordia, Friuli Centrale University Healthcare Hospital of Udine, 33100 Udine, Italy; 3 Fondazione Italiana Fegato ETS—Italian Liver Foundation ETS, 34149 Trieste, Italy

**Keywords:** cutaneous melanoma, skin cancer, *IL1B*, Interleukin-1beta, rs1143634, IL-1 family, single-nucleotide polymorphism, innate immunity, inflammation, case–control study

## Abstract

**Background:** Immune modulation is central to cutaneous melanoma, and antitumor immune responses may be influenced by host genetic background. This study investigated the association between the interleukin-1β gene (*IL1B*) exon 5 synonymous single-nucleotide polymorphism rs1143634 (+3954 C>T) and cutaneous melanoma in a Northeast Italian case–control cohort. **Methods:** The study included 133 Caucasian patients with cutaneous melanoma and 945 healthy controls from Northeast Italy. The rs1143634 polymorphism was genotyped by PCR-restriction fragment length polymorphism (PCR-RFLP). **Results:** Compared with healthy controls, melanoma patients showed higher frequencies of the rs1143634 T allele [27.8% vs. 20.3%; odds ratio (OR) = 1.52, 95% confidence interval (CI) = 1.13–2.03, *p* = 0.005] and CT genotype (43.6% vs. 31.6%; OR = 1.67, CI = 1.16–2.42, *p* = 0.006), whereas the CC genotype was less frequent (50.4% vs. 63.9%; OR = 0.57, CI = 0.40–0.83, *p* = 0.003). TT + CT genotypes were more frequent among non-metastatic melanoma cases than controls (OR = 2.23, CI = 1.37–3.64, *p* = 0.001), but the direct comparison between metastatic and non-metastatic cases was not statistically significant. Among melanoma patients, TT + CT carriers showed an inverse association with Stage IV disease (OR = 0.38, CI = 0.15–0.94, *p* = 0.036) and a positive association with upper-limb melanoma (OR = 9.10, CI = 1.11–75.0, *p* = 0.040); these subgroup findings were exploratory because of small numbers and wide confidence intervals. **Conclusions:** These preliminary findings suggest a possible association between *IL1B* rs1143634 T allele carriage and cutaneous melanoma susceptibility in this cohort. The Stage IV and upper-limb observations should be considered hypothesis-generating. Larger independent studies, correction-aware statistical designs, cytokine or expression measurements, and functional validation are needed to confirm these observations and clarify their biological relevance.

## 1. Introduction

Developing more personalized approaches to cutaneous melanoma prevention and management remains a major challenge in current research [1,2]. Improved understanding of immune modulation in cancer development may enhance patient management and cancer prevention through tailored therapeutic strategies [1,2,3]. In advanced melanoma, immune-based therapies have improved overall survival in a subset of patients; however, many patients still experience disease progression [2,3,4]. Therefore, identifying host factors that may influence melanoma susceptibility remains a priority, and molecular genetic studies are relevant within a precision-medicine framework [1,2,3,4,5,6,7,8,9].

A growing body of evidence indicates that multiple factors contribute to melanoma risk. Host factors include fair skin, number of nevi, genetic background, inflammatory responses, and oxidative stress. Environmental and lifestyle factors include ultraviolet (UV) radiation exposure, especially intermittent sun exposure and sunburns, indoor tanning, smoking, obesity, and selected pollutants [3,5,8,10,11,12,13]. Some candidate genes have been identified through studies on hereditary predisposition [8,9], vitamin D-related pathways [5,6], and innate immunity [7].

Despite recent advances, the role of immune modulation in cutaneous melanoma susceptibility and aggressiveness remains incompletely understood [2,3,4,14]. Emerging evidence suggests that interleukin-1β (IL-1β) may act as a driver of tumor initiation and progression [14,15,16].

IL-1β is a pleiotropic cytokine and a key mediator of inflammation belonging to the interleukin-1 family, which includes 11 cytokines and 11 receptors involved in orchestrating innate immune responses [16]. IL-1β plays a central role in several physiological and pathological processes, modulating immune responses to infection, tissue injury, and tumors [7,14,15,16,17]. Notably, the IL-1 pro-inflammatory cytokines (including IL-1β and IL-1α) can exert dual roles in cancer, contributing to either tumor promotion or suppression, including metastasis [14,15,16].

IL-1β activity is complex and finely modulated. Upon binding to the type 1 IL-1 receptor (IL-1R1), IL-1β induces kinase cascades that activate several factors, including nuclear factor kappa B (NF-κB) and cyclooxygenase-2 (COX-2), two biomarkers implicated in cancer [14,16]. The IL-1 family of cytokines and receptors is involved in a broad spectrum of immunological responses, including lymphocyte activation [16]. Moreover, IL-1β promotes the production of interleukin-8 (IL-8/CXCL8), leading to recruitment and activation of neutrophils and macrophages [14,15,16,17]. IL-1β activity is tightly modulated by the interleukin-1 receptor antagonist (IL-1RA), which binds to IL-1 receptors without triggering signaling. IL-1RA is encoded by the *IL1RN* gene, whose polymorphisms may modulate the competing activity of IL-1RA and thereby influence IL-1β action [7,16].

Increasing evidence indicates that genetic polymorphisms in IL-1 family genes influence susceptibility to inflammatory and infectious diseases and cancer [7,16,18,19]. In this context, a previous Italian study has shown that a variable number of tandem repeats (VNTR) polymorphism in *IL1RN* is associated with cutaneous melanoma risk [7].

The human *IL1B* gene is located on chromosome 2 (2q13-2q21) [20]. Several studies and meta-analyses [21,22] have investigated the role of the *IL1B* rs1143634 polymorphism (previously referred to as +3954, +3953 or TaqI polymorphism), located in exon 5, in relation to various diseases and multiple cancers, particularly gastric cancer, breast cancer, multiple myeloma, intrahepatic cholangiocarcinoma, and lung cancer [21,22,23,24]. However, its role across different tumor types remains incompletely defined, and further studies in cancers of different tissue origins are warranted [21,22].

Currently, understanding the contribution of IL-1 family polymorphisms to cancer poses an important challenge, particularly in melanoma research [7,14,15,16,21,22].

To date, the *IL1B* rs1143634 polymorphism has not been investigated in relation to cutaneous melanoma. In this study, we evaluated the frequency of the *IL1B* rs1143634 polymorphism alleles and genotypes in a Caucasian population from Northeast Italy and their association with cutaneous melanoma (M), including comparisons between metastatic melanoma (MetM, Stage III and IV) and non-metastatic melanoma (NMetM, Stage I and II) cases. Melanoma patients were compared with a large cohort of healthy controls.

## 2. Materials and Methods

### 2.1. Population

Cutaneous melanoma patients and healthy controls were recruited and clinically evaluated at the Dermatology Clinic of the University Hospital of Udine [7]. Diagnostic procedures were performed according to standard clinical protocols. The study protocol was approved by the Institutional Ethics Committee of the University of Udine and was conducted in accordance with the Declaration of Helsinki. All participants provided written informed consent before enrollment [7].

In this case–control study, 133 unrelated patients (age range: 31–89 years), either hospitalized or outpatients, of both sexes, with a documented diagnosis of cutaneous melanoma, were consecutively enrolled. The control group consisted of 945 asymptomatic healthy individuals of both sexes (age range: 30–90 years), matched for age, sex, and ancestry with melanoma patients. Among healthy controls, 458 subjects (of which 148 were healthy non-athlete males and 310 were healthy non-athlete females without bacterial vaginosis) had been previously included in earlier studies [18,25]. Genotyping for the previously collected controls was performed using the same PCR-RFLP protocol in the same laboratory, although the possibility of residual batch or selection effects cannot be fully excluded and is addressed as a study limitation.

Inclusion criteria for all participants were: Caucasian origin, residence in Friuli-Venezia Giulia (FVG) Region, an Italian territory at the border with Austria and Slovenia, and at least two Italian grandparents born in the FVG Region (or in the Austro-Hungarian territory prior to World War I), as described [5,6,7]. Ancestry was based on self-reported family history; genetic ancestry markers were not used. Exclusion criteria for healthy controls included any history of malignant or benign tumors and major acute or chronic diseases, including severe autoimmune conditions such as type 1 diabetes [5,6,7,18]. This strict definition of healthy controls may have generated a selected control group, and this issue is considered in the limitations. The healthy control group included 475 males and 470 females.

Cutaneous melanoma diagnosis was based on histopathological examination following surgical excision of suspicious lesions identified through clinical and dermoscopic evaluation. Tumor staging was determined according to clinical, histological, and radiological criteria, as described [7,26]. Metastatic melanoma (MetM) comprised Stage III regional metastatic disease and Stage IV distant metastatic disease; non-metastatic melanoma (NMetM) comprised Stage I and II disease. Only cutaneous, not mucosal, melanomas were included. In patients with multiple melanomas, the index melanoma was defined as the primary melanoma with the highest T category and was used for the present analyses [7,26]. All non-metastatic patients were followed for at least 5 years after the first melanoma diagnosis to assess the absence of metastasis development.

Each participant completed a structured questionnaire collecting demographic data, medical history, family history of cancer, and smoking habits, as previously described [5,6,7]. Skin phototype was assessed according to the Fitzpatrick classification criteria [27]. Body mass index (BMI) was calculated as weight (kg) divided by height squared (m^2^); BMI values ≥ 30 kg/m^2^ were classified as obesity.

### 2.2. Genetic Analysis of IL1B rs1143634 (+3954 C>T) Polymorphism

The *IL1B* rs1143634 polymorphism was genotyped as previously described [18,25]. Genomic DNA was extracted from ethylenediaminetetraacetic acid (EDTA)-treated peripheral venous blood samples [25]. The polymorphism located in exon 5 was analyzed using the following primers: 5′-GTTGTCATCAGACTTTGACC-3′ and 5′-TTCAGTTCATATGGACCAGA-3′, as described [18,25,28]. The rs1143634 SNP is a synonymous substitution (Phe105Phe), in which the codon TTC is mutated to TTT [28]. PCR-amplified products were digested with the TaqI restriction enzyme, generating fragments of 249 bp (allele T) and fragments of 135 bp plus 114 bp (allele C) [18]. Based on fragment patterns, genotypes were classified as follows: TT (one fragment, 249 bp), CT (three fragments: 249, 135, and 114 bp), and CC (two fragments: 135 and 114 bp). All genotyping procedures were performed with laboratory personnel blinded to clinical and demographic data.

### 2.3. Statistical Analysis

Continuous variables were expressed as mean ± standard deviation (SD) and compared using Student’s *t*-test for independent samples after inspection of distributional assumptions; variables with sparse observations or skewed distributions were interpreted cautiously. Deviations from the Hardy–Weinberg equilibrium (HWE) of genotype distributions were assessed in melanoma patients and healthy controls separately, using exact tests because of the small TT genotype count. Odds ratios (ORs) and 95% confidence intervals (CIs) were calculated for categorical variables. Statistical significance was evaluated using two-sided Pearson’s chi-square or Fisher’s exact test, as appropriate. The primary genetic model was a dominant model comparing T allele carriers (TT + CT genotypes) with CC homozygotes, used as the reference group [25].

A two-sided *p*-value ≤ 0.050 was considered statistically significant, while *p* ≤ 0.100 was considered indicative of a trend and denoted by ^. Analyses of Stage IV disease, anatomical localization, and the many clinicopathological variables were considered exploratory and hypothesis-generating because no formal multiple-testing correction was applied. Multivariable logistic regression among melanoma patients evaluated the association between *IL1B* rs1143634 T allele carriage and selected melanoma clinical features after adjustment for age at first melanoma diagnosis and sex. Due to the limited number of events for some outcomes, the adjusted models were intentionally restricted to these two variables to avoid overfitting. A multivariable melanoma-versus-control model including phototype, nevi, sunburn history, indoor tanning, smoking, BMI, and other host factors could not be performed because these variables were not available in a harmonized format for all controls. Statistical analyses were performed using SPSS for Windows (SPSS Inc., Chicago, IL, USA), version 28 software.

## 3. Results

All 1078 study participants (133 cutaneous melanoma patients and 945 healthy controls) were Italian Caucasian residents in Northeast Italy. The mean ± SD age of melanoma patients was 60.9 ± 12.8 years, with no significant difference compared with that of healthy controls (60.0 ± 15.9 years). Cases and controls did not differ for male sex (75/133 versus 475/945, OR = 1.28, CI = 0.89–1.84, *p* = 0.186).

### 3.1. Comparison of IL1B rs1143634 (+3954 C>T) Genotypes in Cutaneous Melanoma Patients and Healthy Controls (Table 1)

In the overall 1078 study participants, the most frequent allele was C (78.8%, 1699/2156), followed by the T allele (21.2%, 457/2156). Genotype frequencies were as follows: TT 6.0%, CT 43.6%, and CC 50.4% in melanoma patients, and TT 4.4%, CT 31.6%, and CC 63.9% in healthy subjects. Genotype distributions were in Hardy–Weinberg equilibrium in both melanoma patients (exact *p* = 0.392) and healthy controls (exact *p* = 0.546).

The homozygous TT genotype did not differ significantly between groups, as illustrated in Table 1. In contrast, the heterozygous CT genotype was significantly more frequent in melanoma patients than in controls (OR = 1.67, *p* = 0.006). The homozygous CC genotype was less frequent among melanoma patients than controls (OR = 0.57, *p* = 0.003).

**Table 1:** Genotype and allele frequencies of the *IL1B* rs1143634 polymorphism in 133 cutaneous melanoma patients compared with 945 healthy controls.

	Melanoma Patients (n = 133)	Healthy Controls (n = 945)	OR (CI), *p* Value, Melanoma vs. Healthy Subjects
*IL1B* genotype			
TT	8 (6.0%)	42 (4.4%)	1.38 (0.63–3.00), 0.422
CT	58 (43.6%)	299 (31.6%)	**1.67 (1.16–2.42), 0.006**
CC	67 (50.4%)	604 (63.9%)	**0.57 (0.40–0.83), 0.003**
TT + CT	66 (49.6%)	341 (36.1%)	**1.74 (1.21–2.51), 0.003**
*IL1B* allele			
Allele T	74/266 (27.8%)	383/1890 (20.3%)	**1.52 (1.13–2.03), 0.005**
Allele C	192/266 (72.2%)	1507/1890 (79.7%)	**0.66 (0.49–0.88), 0.005**

Differences between groups were evaluated by OR and CI for categorical variables. Statistically significant differences are indicated in bold.

Carriers of the T allele (TT + CT genotypes) were more frequent among melanoma patients than among healthy controls (OR = 1.74, *p* = 0.003). Consistently, the T allele frequency was higher in melanoma patients (27.8%) than in healthy subjects (20.3%) (OR = 1.52, *p* = 0.005).

### 3.2. Comparison of IL1B rs1143634 Genotypes in Metastatic (MetM) and Non-Metastatic (NMetM) Cutaneous Melanoma Patients (Table 2)

No significant differences in genotype distribution were observed between MetM and NMetM patients, as illustrated in Table 2. MetM included Stage III regional metastatic disease and Stage IV distant metastatic disease, whereas NMetM included Stage I and II disease without metastasis after follow-up. When comparing 63 MetM patients with healthy controls, no statistically significant differences were found, although the TT + CT genotypes were slightly more frequent in MetM patients (42.9%) than in healthy subjects (36.1%) (OR = 1.33, *p* = 0.281). Therefore, the study did not detect a statistically significant difference between metastatic and non-metastatic melanoma cases.

**Table 2:** Genotype and allele frequencies of *IL1B* rs1143634 polymorphism comparisons between 63 metastatic melanoma (MetM) patients, 70 non-metastatic melanoma (NMetM) patients, and 945 healthy controls.

	MetM (n = 63)	NMetM (n = 70)	OR (CI), *p* Value, MetM vs. NMetM	OR (CI), *p* Value, MetM vs. Healthy Controls (n = 945)	OR (CI), *p* Value, NMetM vs. Healthy Controls (n = 945)
*IL1B* genotype					
TT	3 (4.8%)	5 (7.1%)	0.65 (0.15–2.84), 0.567	1.07 (0.32–3.57), 0.906	1.65 (0.63–4.32), 0.305
CT	24 (38.1%)	34 (48.6%)	0.65 (0.33–1.30), 0.225	1.33 (0.79–2.25), 0.289	**2.04 (1.25–3.33), 0.004**
CC	36 (57.1%)	31 (44.3%)	1.68 (0.84–3.33), 0.140	0.75 (0.45–1.26), 0.281	**0.45 (0.27–0.73), 0.001**
TT + CT	27 (42.9%)	39 (55.7%)	0.60 (0.30–1.18), 0.140	1.33 (0.79–2.23), 0.281	**2.23 (1.37–3.64), 0.001**
*IL1B* allele					
Allele T	30/126 (23.8%)	44/140 (31.4%)	0.68 (0.40–1.17), 0.167	1.23 (0.80–1.88), 0.340	**1.80 (1.24–2.62), 0.002**
Allele C	96/126 (76.2%)	96/140 (68.6%)	1.47 (0.85–2.53), 0.167	0.81 (0.53–1.24), 0.340	**0.55 (0.38–0.81), 0.002**

Differences between groups were evaluated by OR and CI for categorical variables. Statistically significant differences are indicated in bold.

In contrast, comparison of 70 NMetM patients with healthy controls showed that the CT genotype was associated with increased odds (OR = 2.04, *p* = 0.004) and the TT + CT genotypes were also associated with approximately two-fold increased odds (OR = 2.23, *p* = 0.001), while the CC genotype was less frequent in NMetM patients than in controls (OR = 0.45, *p* = 0.001). This comparison should not be interpreted as evidence of a differential effect between MetM and NMetM because the direct MetM-versus-NMetM comparison was not statistically significant.

Frequencies of alleles T and C did not differ significantly between MetM and NMetM groups or between MetM patients and healthy subjects, while the T allele was more frequent (OR = 1.80, *p* = 0.002), and the C allele was less frequent (OR = 0.55, *p* = 0.002) in NMetM patients than in healthy controls (Table 2).

### 3.3. Demographic and Clinical Characteristics of Melanoma Patients by IL1B TT + CT Genotypes and CC Genotype (Table 3)

The main demographic and clinical characteristics of the 133 melanoma patients are summarized in Table 3. Differences between T allele carriers (TT + CT genotypes, n = 66) and CC homozygotes (n = 67) were examined among melanoma patients.

**Table 3:** Comparison of clinical characteristics between the two genetic subgroups of *IL1B* rs1143634 polymorphism, TT + CT genotypes (n = 66) versus CC genotypes (n = 67) among 133 melanoma patients.

Characteristic	TT + CT Genotypes (n = 66)	CC Genotype (n = 67)	OR (CI) TT + CT vs. CC	*p* Value TT + CT vs. CC
Age < 50 years at study enrollment, n (%)	12 (18.2)	17 (25.4)	0.65 (0.28–1.50)	0.317
Age at study enrollment, years, mean ± SD	62.5 ±12.9	59.3 ± 12.7	-	0.151
Age < 50 years at melanoma diagnosis	20 (30.3)	27 (40.3)	0.64 (0.31–1.32)	0.229
Age at melanoma diagnosis, years, mean ± SD	55.8 ± 13.5	52.6 ± 13.9	-	0.186
Females, n (%)	32 (48.5)	26 (38.8)	1.48 (0.75–2.96)	0.261
Males, n (%)	34 (51.5)	41 (61.2)	0.67 (0.34–1.34)	0.261
All grandparents born in FVG	45 (68.2)	51 (76.1)	0.67 (0.31–1.44)	0.308
BMI ≥ 30 kg/m^2^, n (%)	12 (18.2)	10 (14.9)	1.27 (0.51–3.17)	0.614
Present smoker, n (%)	4 (6.1)	8 (11.9)	0.48 (0.14–1.66)	0.245
Ever smoker, n (%)	33 (50.0)	32 (47.8)	1.09 (0.55–2.16)	0.796
Phototype 1 and 2, n (%)	40 (60.6)	37 (55.2)	1.25 (0.63–2.49)	0.530
Nevi ≥ 50, n (%)	30 (45.5)	36 (53.7)	0.72 (0.36–1.42)	0.340
Low/no tanner, n (%)	36 (54.5)	38 (56.7)	0.92 (0.46–1.82)	0.801
Burns over 5 lifelong, n (%)	35 (53.0)	36 (53.7)	0.97 (0.49–1.92)	0.935
Indoor tanning ≥ 1 ever, n (%)	12 (18.2)	18 (26.9)	0.60 (0.26–1.38)	0.233
MetM, n (%)	27 (40.9)	36 (53.7)	0.60 (0.30–1.18)	0.140
Stage I, n (%)	30 (45.5)	22 (32.8)	1.70 (0.84–3.44)	0.137
Stage II, n (%)	9 (13.6)	9 (13.4)	1.02 (0.38–2.75)	0.973
Stage III, n (%)	19 (28.8)	18 (26.9)	1.10 (0.51–2.35)	0.805
Stage IV, n (%)	8 (12.1)	18 (26.9)	**0.38 (0.15–0.94)**	**0.036**
Trunk, n (%)	34 (51.5)	41 (61.2)	0.67 (0.34–1.34)	0.261
Upper limb, n (%)	8 (12.1)	1 (1.5)	**9.10 (1.11–75.0)**	**0.040**
Lower limb, n (%)	16 (24.2)	12 (17.9)	1.47 (0.63–3.40)	0.372
Hands/feet, n (%)	3 (4.5)	5 (7.5)	0.59 (0.14–2.58)	0.483
Head/neck, n (%)	5 (7.6)	8 (11.9)	0.60 (0.19–1.95)	0.400
Superficial spreading, n (%)	34 (51.5)	36 (53.7)	0.91 (0.46–1.81)	0.798
Nodular, n (%)	24 (36.4)	21 (31.3)	1.25 (0.61–2.57)	0.541
Acral lentiginous, n (%)	2 (3.0)	3 (4.5)	0.67 (0.11–4.12)	0.663
Lentigo maligna, n (%)	1 (1.5)	1 (1.5)	1.02 (0.06–16.6)	0.991
Spitzoide, n (%)	2 (3.0)	3 (4.5)	0.67 (0.11–4.12)	0.663
Other histologic subtypes, n (%)	4 (6.1)	5 (7.5)	0.80 (0.21–3.12)	0.748
Breslow thickness, mm, mean ± SD	1.84 ± 1.63	2.25 ± 2.05	-	0.202
Ulceration, n (%)	23 (34.8)	28 (41.8)	0.74 (0.37–1.50)	0.411
Mitosis ≥ 1 per mm^2^, n (%)	44 (67.7) ^b^	39 (59.1) ^c^	1.45 (0.71–2.96)	0.308
Regression, n (%)	9 (13.8) ^b^	11 (16.7) ^c^	0.80 (0.31–2.09)	0.654
Brisk positive TILs ^a^, n (%)	19 (29.2) ^b^	19 (28.8) ^c^	1.02 (0.48–2.17)	0.956
Non-brisk TILs ^a^, n (%)	22 (33.8) ^b^	25 (37.9) ^c^	0.84 (0.41–1.72)	0.631
TILs ^a^ absence, n (%)	23 (35.4) ^b^	22 (33.3) ^c^	1.10 (0.53–2.25)	0.805
More than 1 melanoma, n (%)	9 (13.6)	12 (17.9)	0.72 (0.28–1.85)	0.500
Additional non-melanoma skin cancer, n (%)	10 (15.2)	12 (17.9)	0.82 (0.33–2.05)	0.669
Additional non-skin cancer, n (%)	16 (24.2)	13 (19.4)	1.33 (0.58–3.04)	0.500
Melanoma familiarity, n (%)	9 (13.6)	9 (13.4)	1.02 (0.38–2.75)	0.973
Dead ≤ 5 years from first diagnosis	7 (10.6)	11 (16.4)	0.60 (0.22–1.67)	0.331

Differences between groups were evaluated by OR and CI for categorical variables and by Student’s *t*-test for continuous variables. ^a^ TILs, tumor-infiltrating lymphocytes. ^b^ Data were available for 65 patients. ^c^ Data were available for 66 patients. Significant differences are indicated in bold.

The proportions of melanoma patients aged < 50 years at study entry and at first melanoma diagnosis were lower for TT + CT genotype carriers, but the differences were not statistically significant.

The proportion of patients with Stage IV melanoma was lower in TT + CT than in CC carriers (12.1% versus 26.9%, OR = 0.38, *p* = 0.036). Conversely, melanoma localization in the upper limbs was more frequent among TT + CT than CC carriers (12.1% versus 1.49%, OR = 9.10, *p* = 0.040). Because these analyses involved small subgroups and many comparisons, these findings should be regarded as exploratory. In a sensitivity analysis based on the anatomical definition of the upper extremity, one patient (CC genotype) with melanoma localized on the hand was added to the upper-limb group. This attenuated the association (12.1% TT + CT versus 2.98% CC carriers; OR = 4.48, CI = 0.91–22.0, *p* = 0.064 ^).

No additional significant differences were observed between the TT + CT and CC genotype groups.

The study comprised 26 patients with Stage IV melanoma; their genotype distribution was: TT 3.8% (1/26), CT 26.9% (7/26), and CC 69.2% (18/26). In the remaining 107 melanoma patients, frequencies were: TT 6.5% (7/107), CT 47.7% (51/107), and CC 45.8% (49/107). Comparative analysis between these two groups showed: TT, OR = 0.57, CI = 0.07–4.86, *p* = 0.608; CT, OR = 0.40, CI = 0.16–1.04, *p* = 0.061 ^; and CC, OR = 2.66, CI = 1.07–6.65, *p* = 0.036. These subgroup results were not corrected for multiple testing and should be considered hypothesis-generating.

When Stage IV melanoma cases were compared with healthy controls, no significant differences in genotype distribution were observed: TT, OR = 0.86, CI = 0.11–6.50, *p* = 0.884; CT, OR = 0.80, CI = 0.33–1.91, *p* = 0.610; and CC, OR = 1.27, CI = 0.55–2.95, *p* = 0.578.

### 3.4. Multivariable Logistic Regression Analysis Among Melanoma Patients

In multivariable logistic regression analyses, after adjusting for age at first melanoma diagnosis and sex and comparing TT + CT genotypes with CC homozygotes used as the reference group, carriage of the *IL1B* rs1143634 TT + CT genotypes was not significantly associated with metastatic melanoma status. The crude OR for metastatic versus non-metastatic melanoma was 0.60, CI = 0.30–1.18, *p* = 0.140, and remained non-significant after adjustment for age at first melanoma diagnosis and sex, adjusted OR = 0.62, CI = 0.31–1.25, *p* = 0.180. Adjustment was limited to age and sex because additional melanoma risk factors were not available in a harmonized format for all controls and because the number of events was limited for subgroup outcomes.

TT + CT carriage was inversely associated with Stage IV disease. The crude OR was 0.38, CI = 0.15–0.94, *p* = 0.036, and the association remained similar after adjustment for age at first diagnosis and sex, aOR = 0.38, CI = 0.15–0.96, *p* = 0.041. TT + CT carriage was also associated with upper-limb melanoma localization. The crude OR was 9.10, CI = 1.11–74.98, *p* = 0.040, and the age- and sex-adjusted aOR was 11.45, CI = 1.33–98.97, *p* = 0.027. When hand localization was included with upper-limb melanomas, the association was attenuated but remained directionally consistent, with an age- and sex-adjusted aOR of 5.51, CI = 1.07–28.35, *p* = 0.041. These adjusted subgroup analyses were exploratory and imprecise, as indicated by the small numbers and wide confidence intervals.

### 3.5. Clinical Characteristics of Patients with Upper-Limb Melanoma (Table 4)

As illustrated in Table 4, when comparing nine melanoma patients with upper-limb tumors with the remaining 124 melanoma patients, the only nominally significant findings were a higher frequency of the CT heterozygous genotype (OR = 5.01, *p* = 0.050) and a lower frequency of the CC homozygous genotype (OR = 0.11, *p* = 0.040). Given the very small number of upper-limb cases and the number of tested variables, these findings should be interpreted as exploratory. The proportion of patients in Stage II showed a trend toward higher frequency (OR = 3.63, *p* = 0.089 ^) among participants with melanoma in the upper limbs compared to other sites. None of the patients with upper-limb localization had multiple melanomas, but this finding was not statistically significant.

**Table 4:** Comparison of clinical characteristics between 9 patients with upper-limb melanoma and 124 patients with other melanoma localizations, among a total of 133 melanoma patients.

Characteristic	Upper-Limb Melanoma (n = 9)	Other Body Sites Melanoma (n = 124)	OR (CI)	*p* Value
TT genotype, n (%)	1 (11.1)	7 (5.6)	2.09 (0.23–19.1)	0.514
CT genotype, n (%)	7 (77.8)	51 (41.1)	**5.01 (1.00–25.1)**	**0.050**
CC genotype, n (%)	1 (11.1)	66 (53.2)	**0.11 (0.01–0.90)**	**0.040**
Age < 50 years, at study enrollment n (%)	3 (33.3)	26 (21.0)	1.88 (0.44–8.05)	0.392
Age at study enrollment, years, mean ± SD	54.1 ± 12.5	61.4 ± 12.8	-	0.101
Age < 50 years at melanoma diagnosis	5 (55.6)	42 (33.9)	2.44 (0.62–9.57)	0.201
Age at melanoma diagnosis, years, mean ± SD	46.9 ± 11.7	54.7 ± 13.8	-	0.099 ^
Females, n (%)	5 (55.6)	53 (42.7)	1.67 (0.43–6.54)	0.458
Males, n (%)	4 (44.4)	71 (57.3)	0.60 (0.15–2.33)	0.458
All grandparents born in FVG	6 (66.7)	90 (72.6)	0.76 (0.18–3.19)	0.703
BMI ≥ 30 kg/m^2^, n (%)	2 (22.2)	20 (16.1)	1.49 (0.29–7.68)	0.637
Present smoker, n (%)	2 (22.2)	10 (8.1)	3.26 (0.60–17.8)	0.173
Ever smoker, n (%)	4 (44.4)	61 (49.2)	0.83 (0.21–3.22)	0.783
Phototype 1 and 2, n (%)	7 (77.8)	70 (56.5)	2.70 (0.54–13.5)	0.227
Nevi ≥ 50, n (%)	4 (44.4)	62 (50.0)	0.80 (0.21–3.12)	0.748
Low/no tanner, n (%)	7 (77.8)	67 (54.0)	2.98 (0.59–14.9)	0.184
Burns over 5, n (%)	4 (44.4)	67 (54.0)	0.68 (0.17–2.66)	0.580
Indoor tanning ≥ 1 ever, n (%)	2 (22.2)	28 (22.6)	0.98 (0.19–4.98)	0.980
MetM, n (%)	4 (44.4)	59 (47.6)	0.88 (0.23–3.44)	0.856
Stage I, n (%)	2 (22.2)	50 (40.3)	0.42 (0.08–2.12)	0.295
Stage II, n (%)	3 (33.3)	15 (12.1)	3.63 (0.82–16.1)	0.089 ^
Stage III, n (%)	3 (33.3)	34 (27.4)	1.32 (0.31–5.59)	0.703
Stage IV, n (%)	1 (11.1)	25 (20.2)	0.49 (0.06–4.14)	0.516
Superficial spreading, n (%)	6 (66.7)	64 (51.6)	1.87 (0.45–7.83)	0.389
Nodular, n (%)	3 (33.3)	42 (33.9)	0.98 (0.23–4.10)	0.974
Acral lentiginous, n (%)	0 (-)	5 (4.0)	1.14 (0.06–22.3)	0.929
Lentigo maligna, n (%)	0 (-)	2 (1.6)	2.58 (0.12–57.7)	0.550
Spitzoide, n (%)	0 (-)	5 (4.0)	1.14 (0.06–22.3)	0.929
Other histologic subtypes, n (%)	0 (-)	9 (7.3)	0.64 (0.03–11.9)	0.764
Breslow thickness, mm, mean ± SD	2.31 ± 1.57	2.03 ± 1.88	-	0.659
Ulceration, n (%)	5 (55.6)	46 (37.1)	2.12 (0.54–8.29)	0.280
Mitosis ≥ 1 per mm^2^, n (%)	7 (77.8)	76 (62.3) ^b^	2.12 (0.42–10.6)	0.362
Regression, n (%)	0 (-)	20 (16.4) ^b^	0.26 (0.01–4.70)	0.364
Brisk positive TILs ^a^, n (%)	3 (33.3)	35 (28.7) ^b^	1.24 (0.29–5.25)	0.767
Non-brisk TILs ^a^, n (%)	4 (44.4)	43 (35.2) ^b^	1.47 (0.37–5.76)	0.581
TILs ^a^ absence, n (%)	2 (22.2)	43 (35.2) ^b^	0.52 (0.10–2.64)	0.434
More than 1 melanoma, n (%)	0 (-)	21 (16.9)	0.25 (0.01–4.52)	0.350
Additional non-melanoma skin cancer, n (%)	1 (11.1)	21 (16.9)	0.61 (0.07–5.17)	0.653
Additional non-skin cancer, n (%)	1 (11.1)	28 (22.6)	0.43 (0.05–3.57)	0.434
Melanoma familiarity, n (%)	1 (11.1)	17 (13.7)	0.79 (0.09–6.69)	0.826
Dead ≤ 5 years from first diagnosis	1 (11.1)	17 (13.7)	0.79 (0.09–6.69)	0.826

Differences between groups were evaluated by OR and CI for categorical variables and by Student’s *t*-test for continuous variables. ^a^ TILs, tumor-infiltrating lymphocytes; ^b^ Data were available for 122 patients. Significant differences are indicated in bold, and tendencies were evidenced with superscript ^.

## 4. Discussion

This study, performed in a Northeast Italian case–control cohort, represents the first investigation of the *IL1B* rs1143634 (+3954 C>T) polymorphism in cutaneous melanoma. T allele (TT + CT genotypes) carriage was 1.74-fold more frequent in melanoma patients than in healthy controls, suggesting a possible association with melanoma susceptibility in this population. In contrast, the direct comparison between metastatic melanoma (MetM) and non-metastatic melanoma (NMetM) was not statistically significant; therefore, these data do not establish a differential effect of rs1143634 on metastatic status. Among melanoma patients, the TT + CT genotype group was less frequent, whereas the CC genotype was more frequent (OR = 2.66, *p* = 0.036) in Stage IV melanoma patients than in non-Stage IV patients. Moreover, the TT + CT genotype group was more frequent (OR = 9.10, *p* = 0.040) in patients with melanoma localized in the upper limbs than in other anatomical sites. However, Stage IV and upper-limb observations have to be retained as exploratory, hypothesis-generating findings because they are based on small subgroups, wide confidence intervals, and multiple uncorrected comparisons.

Genotype distributions in this cohort were: TT 6.0%, CT 43.6%, CC 50.4% in all 133 melanoma patients; TT 7.1%, CT 48.6%, CC 44.3% in NMetM; TT 4.8%, CT 38.1%, CC 57.1% in MetM; and TT 4.4%, CT 31.6%, CC 63.9% in healthy controls. These findings are broadly consistent with previous Italian data from healthy subjects, including 406 healthy women from Northeast Italy showing genotype frequencies of TT 3.7%, CT 33.7%, CC 62.6% [18], and 546 healthy controls from Tuscany (Central Italy) showing TT 6.0%, CT 33.3%, CC 60.6% [29]. The allele frequencies that we found in the healthy Italian population (79.7% C allele and 20.3% T allele) were closely comparable to those reported in European populations (79.0% C allele and 21.0% T allele) [30].

### 4.1. Biological Interpretation of IL1B rs1143634

IL-1β is a pleiotropic cytokine with context-dependent roles in cancer biology [14,15,16,31,32]. IL-1β may contribute to tumor-promoting inflammation, angiogenesis, immunosuppression, and tumor-cell invasiveness in some settings, while also participating in host defense and antitumor immunity in others [14,15,16,31,32,33,34,35,36,37,38,39,40,41,42,43]. This duality provides a plausible biological framework for future studies, but it should not be interpreted as direct evidence that rs1143634 modifies IL-1β activity in the patients analyzed here.

The rs1143634 variant is a synonymous SNP; therefore, the C-to-T transition does not alter the encoded amino acid sequence of IL-1β. Previous studies have suggested that this variant, or variants in linkage disequilibrium with it, may be associated with increased IL-1β production in some settings [19,21,44,45]. Of note, a study showed that in patients with coronary heart disease, the rs1143634 T allele was associated with increased C-reactive protein (CRP), an inflammatory marker induced mainly by IL-6, synergized by IL-1β [46].

However, results across diseases and populations have been inconsistent, and the present study did not measure circulating IL-1β, CRP, inflammasome activation, *IL1B* expression, IL-1 receptor signaling, or the inflammatory microenvironment in melanoma tissue. Consequently, the biological mechanism underlying the observed association remains unresolved.

In our cohort of melanoma patients, the TT + CT genotypes did not differ from the CC genotype for main demographic characteristics, sex, or several established melanoma risk factors, such as fair skin or number of nevi. Likewise, the TT + CT genotype group was not associated with known risk factors for severe melanoma, such as tumor thickness, ulceration, and others. The only statistically significant finding was the lower proportion of T allele carrier patients aged < 50 years at study entry; however, this variable was not statistically significant for age < 50 years at first melanoma diagnosis. Larger studies are warranted to assess whether T carriers are less likely to develop melanoma before 50 years of age.

The current data demonstrate a statistical association rather than a functional role of *IL1B* rs1143634 in cutaneous melanoma. The comprehensive effects of the rs1143634 T allele on IL-1β activity may be influenced by interactions with several elements involved in the entire IL-1 pathway.

In studies performed in different cancers, the *IL-1B* rs1143634 CT genotype and T allele have been associated with increased cancer risk, although with heterogeneity with respect to ethnicity [21]. Our data are consistent with some studies on other cancers, particularly gastric cancer [21,22,29]. However, the functional impact of the rs1143634 SNP remains unclear.

The association of the *IL1B* rs1143634 polymorphism with cancer was examined in detail by Jafrin and colleagues [21]; pooled analysis revealed a borderline significant association with increased overall cancer risk when comparing TT versus CT + CC genotypes (OR = 1.14, CI = 1.04–1.25, *p* = 0.006), and when comparing T versus C alleles (OR = 1.08, CI = 1.00–1.17, *p* = 0.039). Subgroup analysis showed that the rs1143634 C>T polymorphism is associated with an increased risk of gastric cancer, breast cancer, and multiple myeloma, while no significant associations were found for lung, colorectal, and prostate cancers [21]. Asian populations showed a significantly higher risk of cancer development compared to other ethnic groups [21].

### 4.2. Metastatic Status and Stage IV Disease

The distinction between melanoma susceptibility, metastatic status, and Stage IV disease is important. In this cohort, MetM included Stage III regional metastatic disease and Stage IV distant metastatic disease, whereas NMetM included Stage I and II disease. T allele carriage was more frequent in NMetM patients than in healthy controls, whereas non-significant differences were observed between MetM and healthy controls, but the direct MetM-versus-NMetM comparison was not significant. Therefore, the difference between a significant NMetM-versus-control comparison and a non-significant MetM-versus-control comparison should not be interpreted as proof of divergent effects. Limited statistical power in the MetM subgroup remains a more conservative interpretation.

The role of IL-1β in cancer is complex and appears to be context-dependent [16,31,32]. Evidence suggests that elevated IL-1β levels may contribute to cancer onset, angiogenesis, and invasiveness of melanoma cells [16]. Human melanoma cells have been shown to constitutively express and secrete IL-1β [32]. Immunohistochemical staining of tissues from patients showed that IL-1β is low or absent in benign nevi and higher in primary and metastatic melanomas [33]. Interestingly, downstream signaling pathways affected by endogenous IL-1 were observed to include reactive oxygen species (ROS) and reactive nitrogen species (RNS), which have roles in melanoma [33]. Furthermore, inactivation of the IL-1β–IL-1R1 system has been shown to arrest melanoma cell growth [33].

Of note, IL-1β and IL-1RA genes appear to be cross-regulated [16,25]. Interestingly, the presence of allele 2 of the *IL1RN* VNTR rs2234663 polymorphism has been associated with enhanced IL-1β production in vitro and with an increased inflammatory response [47]. Notably, a study showed that allele 2 of the *IL1RN* rs2234663 polymorphism is associated with cutaneous melanoma susceptibility (OR = 1.53, *p* = 0.036), without differences between MetM and NMetM patients [7]. Importantly, two different polymorphic alleles—*IL1B* rs1143634 allele T and *IL1RN* rs2234663 allele 2—both potentially promoting increased levels of IL-1β were observed to be associated with cutaneous melanoma susceptibility, but without significant differences between metastatic and non-metastatic patients. Based on the assumption that the rs1143634 polymorphism likely promotes increased IL-1β activity, it might be speculated that a chronically enhanced IL-1-related immune response (due to genetic polymorphisms of the *IL1B* gene) might increase the risk of developing melanoma, without necessarily promoting aggressive disease or even potentially reducing the risk of developing Stage IV melanoma, as observed for the rs1143634 T allele. However, this hypothesis-generating model remains speculative.

The inverse association between TT + CT carriage and Stage IV disease was nominally significant, but it involved only 26 Stage IV cases and was not corrected for multiple testing. It should therefore be interpreted as an exploratory observation requiring replication in larger cohorts with predefined analysis plans.

### 4.3. Upper-Limb Melanoma Localization

The association between TT + CT carriage and upper-limb localization was based on only nine upper-limb melanoma cases and had a very wide confidence interval. This finding may reflect a true site-specific host-genotype effect, but it may also be compatible with chance, given the number of comparisons performed. In addition, individual UV exposure by anatomical site was not measured. For these reasons, the upper-limb result should be considered preliminary and should be tested in independent cohorts designed to address the anatomical site. Nonetheless, this new preliminary site-specific finding suggests an interaction between a specific genetic predisposition and environmental exposure, i.e., that an inflammatory response in skin regions more frequently exposed to sunlight/UV radiation stress might promote melanocyte transformation [48]. We did not find previous evidence of a genetic predisposition for melanoma in the upper limbs [49]. Our observation should be extended and confirmed in larger independent studies.

Upper-extremity melanomas show some specificities. An epidemiological study [49] examined characteristics and survival outcomes of patients with melanoma localized in the upper limbs and shoulders in a large U. S. cohort and compared them with other melanoma body sites. Females were more often affected in the upper extremities compared to other locations, while males were more often affected in other locations than the upper extremities. Women under 50 years were more at risk than men of the same age and appeared to have better survival than men [49].

Of note, a study in Norwegian women [50] on UV radiation exposure and risk of melanoma on different body sites found that recreational sun exposure and indoor tanning were associated with melanoma on the lower limbs—the most common site of melanoma in women—but not with upper-limb melanomas. These findings support a heterogeneous response to radiation by body site. Furthermore, heterogeneity according to the anatomic position of melanoma with respect to oncogenic alterations has been observed [51].

Heterogeneity might also be associated with inflammasome activity. Notably, recent evidence demonstrated that the activation of the NLRP1 (NLR pyrin domain containing 1) inflammasome—the most relevant inflammasome in keratinocytes and a crucial sensor of UVB radiation—supports the development of melanoma by inducing skin inflammation [48,52]. Inflammasome activation activates caspase-1, which then cleaves pro-IL-1β to generate mature IL-1β; thus, it appears likely that genetic factors increasing IL-1β-related inflammation may contribute to the development of skin cancers [21,43,52,53].

Indeed, evidence on the association of sun exposure with melanoma localization is inconsistent across studies [10,50,54].

The upper-limb association with rs1143634 T allele carriage warrants further study, particularly focusing on local effects of the *IL1B* polymorphisms in exposed skin tissues, and should be confirmed in larger studies focused on site specificity of cutaneous melanoma.

### 4.4. Strengths and Limitations

Strengths of the study include a well-defined regional population, a relatively large control group, and standardized genotyping by PCR-RFLP. The geographically and ancestrally restricted design may reduce population heterogeneity, which is relevant because IL-1 family polymorphisms vary by ancestry and region [21,22,25]. Importantly, in this study, detailed information was collected regarding the lifestyle of cutaneous melanoma patients.

The study also has important limitations. First, the melanoma case number was modest, and some subgroup analyses were based on sparse cell counts. Second, multiple clinicopathological comparisons were performed without formal multiple-testing correction; therefore, Table 3 and Table 4 analyses should be regarded as exploratory. Third, control participants were selected as healthy subjects without tumors or major disease, which may have created a selected control group and could have inflated associations. Fourth, 458 controls had been included in previous studies [18,25], and residual differences in recruitment context or batch effects cannot be fully excluded despite the use of the same genotyping protocol. Fifth, ancestry was self-reported and not assessed using genetic ancestry markers. Sixth, major melanoma risk factors, including phototype, number of nevi, sunburn history, indoor tanning, smoking, BMI, and other host factors, were not available in a harmonized format for all controls; consequently, the main case–control analysis could not be fully adjusted for these potential confounders. Finally, no functional validation was performed, and no IL-1β, CRP, transcriptomic, or tissue-microenvironment measurements were available.

Future studies should test *IL1B* rs1143634 polymorphism in larger, independent, and ethnically diverse cohorts with harmonized control demographics and melanoma risk-factor data. Functional studies should determine whether rs1143634 itself is functional or whether it acts as a marker in linkage disequilibrium with another causal variant. Cytokine measurements, CRP levels, *IL1B* expression, melanoma-tissue transcriptomics, and interrogation of public datasets such as TCGA-SKCM could help clarify biological relevance.

## 5. Conclusions

These data suggest a possible association between *IL1B* rs1143634 T allele carriage and cutaneous melanoma susceptibility in a Northeast Italian cohort. The study did not detect a significant difference between metastatic and non-metastatic melanoma cases. The inverse association of TT + CT genotypes with Stage IV disease and the association with upper-limb localization are preliminary, hypothesis-generating observations based on small subgroups and should not be considered clinically actionable. Larger replication studies, correction-aware statistical analysis, functional validation, and cytokine or expression measurements are required before rs1143634 can be considered a melanoma biomarker or incorporated into personalized prevention, prognosis, or therapy models.

## Data Availability

All data from this study are available upon reasonable request.
